# *Stachybotrys* mycotoxins: from culture extracts to dust samples

**DOI:** 10.1007/s00216-016-9649-y

**Published:** 2016-06-02

**Authors:** Ina Došen, Birgitte Andersen, Christopher B. W. Phippen, Geo Clausen, Kristian Fog Nielsen

**Affiliations:** Section for Eukaryotic Biotechnology, Department of Systems Biology, Technical University of Denmark, Søltofts Plads, 2800 Lyngby, Denmark; International Centre for Indoor Environment and Energy, Department of Civil Engineering, Technical University of Denmark, Nils Koppels Allé, 2800 Lyngby, Denmark

**Keywords:** *Stachybotrys*, Mycotoxin, Dust, Spirocyclic drimane, QTOF, QqQ

## Abstract

The filamentous fungus *Stachybotrys chartarum* is known for its toxic metabolites and has been associated with serious health problems, including mycotoxicosis, among occupants of contaminated buildings. Here, we present results from a case study, where an ultra-high performance liquid chromatography-tandem mass spectrometry (UHPLC-MS/MS) method was developed for known and tentatively identified compounds characterized via UHPLC-quadruple time-of-flight (QTOF) screening of fungal culture extracts, wall scrapings and reference standards. The UHPLC-MS/MS method was able to identify 12 *Stachybotrys* metabolites, of which four could be quantified based on authentic standards and a further six estimated based on similarity to authentic standards. Samples collected from walls contaminated by *S. chartarum* in a water-damaged building showed that the two known chemotypes, S and A, coexisted. More importantly, a link between mycotoxin concentrations found on contaminated surfaces and in settled dust was made. One dust sample, collected from a water-damaged room, contained 10 pg/cm^2^ macrocyclic trichothecenes (roridin E). For the first time, more than one spirocyclic drimane was detected in dust. Spirocyclic drimanes were detected in all 11 analysed dust samples and in total amounted to 600 pg/cm^2^ in the water-damaged room and 340 pg/cm^2^ in rooms adjacent to the water-damaged area. Their wide distribution in detectable amounts in dust suggested they could be good candidates for exposure biomarkers.

**Electronic supplementary material:**

The online version of this article (doi:10.1007/s00216-016-9649-y) contains supplementary material, which is available to authorized users.

**Graphical abstract:**
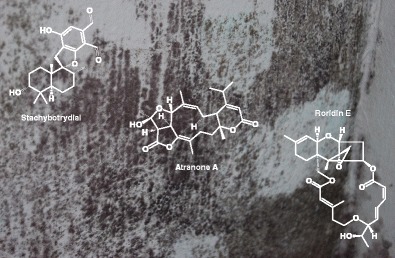
*Stachybotrys* growing on a gypsum board, and some of the compounds it produces

## Introduction

Fungal spores are ubiquitous in indoor environments, and the growth of mould in buildings can often lead to negative health effects such as skin rashes, headaches, dizziness and chronic fatigue of the occupants [1–3]. Although the presence of moulds in the indoor environment is generally undesirable, some species, such as *Stachybotrys chartarum* and *Chaetomium globosum*, are considered more problematic [3]. *S. chartarum* is known for its production of toxic metabolites that have been connected to the extensive occurrence of negative health effects [1, 4] and possibly also linked to idiopathic pulmonary haemosiderosis in babies [5, 6]. *Stachybotrys* has historically been shown to be responsible for severe toxicoses in farm animals fed with contaminated hay [7, 8].

**Fig. 1:**
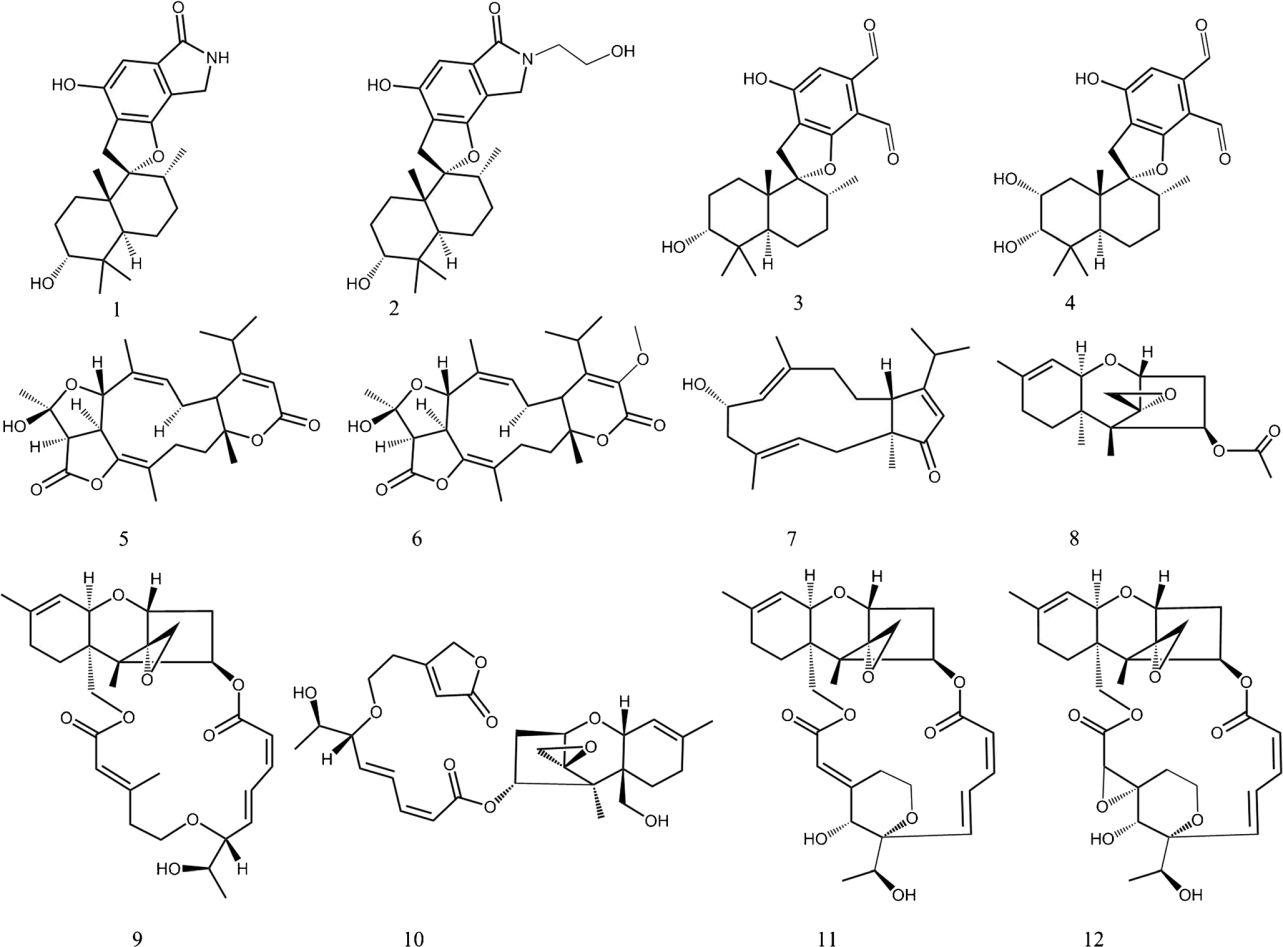
Secondary metabolites and mycotoxins produced by *S. chartarum*: spirocyclic drimanes produced by both chemotypes (*1* stachybotrylactam, *2* stachybotryamide, *3* stachybotrydial, *4* Mer-NF-5003-B), atranones and their precursors characteristic of chemotype A (*5* atranone A, *6* atranone B, *7* and *6* hydroxydolabella-3*E*,7*E*,12-trien-14-one, *8* simple trichothecene trichodermin) and macrocyclic trichothecenes characteristic of chemotype S (*9* roridin E, *10* roridin L2, *11* satratoxin H, *12* satratoxin G)

*S. chartarum* can be found in two chemotypes, S and A, both sharing the same morphology, but differing in some of the metabolites they produce [9]. Chemotype S produces macrocyclic trichothecenes (satratoxins, verrucarins and roridins; Fig. 1), which are some of the most cytotoxic compounds currently known [10]. Chemotype A produces atranones and their precursors, dolabellanes, together with the simple non-macrocyclic trichothecene, trichodermin [9]. Both chemotypes also produce many metabolites belonging to the spirocyclic drimane family [11], in much greater quantity than the trichothecenes and antranones [12–14]. Chemotype A induces highly inflammatory effects both in vivo and in vitro [10, 14, 15], but the causative agents for this have not been revealed yet. It is possible that the observed inflammatory effects are caused by the drimanes, atranones, or other compounds present in both types but that these effects may be concealed in the chemotype S by the highly cytotoxic macrocyclic trichothecenes [14]. Furthermore, *Stachybotrys chlorohalonata* is also found in buildings, but chemically, it can currently not be differentiated from *S. chartarum* chemotype A [9].

*S. chartarum* requires high water activity (0.98) for growth [16], which in practice for the indoor environment means water ingress rather than high levels of condensation. *S. chartarum* is mainly observed on materials with high cellulose content, such as gypsum wallboard, straw, wallpapers and some insulation materials [13, 17]. Spores of *S. chartarum* are produced in sticky slime heads [18] and do not easily become airborne when they are moist, making detection in the air difficult. Hence, exposure mainly occurs from dry, disintegrating spores and mycelium as micro-particles [19–21].

Based on the database Antibase2012, as well as other sources [10, 12, 22–24], there are currently around 140 compounds described from *Stachybotrys* spp. Only one of these compounds, stachybotrylactam, is commercially available whilst satratoxins H and G have also been partially available. A few authors have developed trace analytical methods including these three compounds, based on liquid chromatography-tandem mass spectrometric (LC-MS/MS) detection [25–27]. The macrocyclic trichothecenes can also be analysed by immunochemical methods [28, 29] or via hydrolysis, derivatization and GC-MS [11, 30]. Although a large amount of attention has been placed on the presence of *Stachybotrys* mycotoxins in the indoor environment, attempts to identify these toxics in the dust usually fail, as do attempts to correlate amounts found on contaminated surfaces with amounts found in dust. This failure is especially true in cases where the contaminant is *S. chartarum* chemotype A or *S. chlorohalonata*, as none of existing methods targets atranones or their precursors, the dolabellanes. Furthermore, all the major spirocyclic drimanes, except for stachybotrylactam, are not covered by the existing targeted LC-MS/MS methods.

Earlier analytical methods for *Stachybotrys* metabolites, mainly based on LC separation with UV/vis diode array detection (DAD) [9, 12, 31], clearly showed that drimanes were the dominant compounds in the analysed extracts. In order to separate drimanes from atranones or macrocyclic trichothecenes, samples were purified by normal phase solid phase extraction on PEI silica [12]. The introduction of LC in combination with high-resolution mass spectrometry (HRMS) has allowed both time-of-flight (TOF) and Orbitrap instruments to be used routinely for fungal metabolite profiling [32, 33]. One of the advantages of LC-HRMS over UV/vis is that it is easier to distinguish co-eluting compounds using extracted ion chromatograms [34]. Despite this advantage, many fungal metabolites possess the same elemental composition, and even more specific/accurate identification than HRMS is required to distinguish similar compounds. This greater specificity of identification can be obtained from MS/HRMS either by using standardized MS/HRMS libraries with three fixed fragmentation energies (e.g. 10, 20 and 40 eV as introduced by Agilent Technologies) [35, 36] or more energies, as on ThermoFisher instruments [37].

Our recent work [36] presented the use of MS/HRMS libraries in fungal drug discovery, and we are currently expanding our existing library with *Stachybotrys* metabolites. Agilent Technologies state that fragmentation energies are standardized across their instruments, including triple quadrupole instruments (QqQ). Consequently, we hypothesize that multiple reaction monitoring (MRM) could be predicted directly from the MS/HRMS library, not only including fragment ions but also the collision energies. In culture extracts analysed by UHPLC-DAD-HRMS, we observed that most of the spirocyclic drimanes ionized very well compared to their apparent intensity in the UV/vis chromatogram. Considering that QqQ instruments are typically 5–50-fold more sensitive than HRMS instruments, we hypothesized that by mapping all the major spirocyclic drimanes from *Stachybotrys* cultures and infected material samples and transferring the method to a QqQ instrument, we would stand a greater chance of detecting these in settled dust in *Stachybotrys*-infected buildings than other metabolites such as the trichothecenes.

In this case study, we tested these hypotheses by identifying promising *S. chartarum* biomarkers using UHPLC-QTOF in extracts from pure fungal cultures and cotton tip swabs from infected gypsum wallboards and further transferring detection of these directly to a UHPLC-QqQ method. This method was combined with standards of several macrocyclic trichothecenes, several atranones, one commercially available spirocyclic drimane and the simple trichothecene trichodermin.

## Materials and methods

### Chemicals and standards

All solvents, including water for LC-MS analyses, were LC-MS grade; chemicals were analytical grade and were purchased from Sigma-Aldrich (Steinheim, Germany), if not otherwise stated. Standard of stachybotrylactam was purchased from Enzo (Exeter, UK). All other standards were available in-house, donated by other research groups or purified from different in-house projects [36]. Standards for quantification purposes were weighed and dissolved either in pure acetonitrile (trichodermin (≥95 % pure HPLC)), acetonitrile/water (90:10, *v*/*v*) (roridin E (≥90 % pure HPLC)) or pure methanol (stachybotrylactam (≥95 % pure HPLC); satratoxin H (≥90 % pure HPLC)). Individual stock solutions were kept in −20 °C. Prior to analysis, stock solutions were brought to room temperature, thoroughly mixed and used to prepare multi-analyte standard solutions in triplicates, at five (satratoxin H, roridin E) or six (stachybotrylactame, trichodermin) different levels with concentrations of 10, 100, 300, 1000, 3000 and 10,000 ng/mL.

### Sampling of fungal biomass and settled dust

All samples in this case study were collected from a water-damaged kindergarten in the Greater Copenhagen area. A broken concealed water pipe in a ground floor bathroom had resulted in fungal contamination in the internal structures of the wall (>80 % of the surface covered with fungal growth). The whole ground floor was closed off due to the high number of children (>50 %) reporting sickness.

The wall was opened and fungal biomass samples were collected from the gypsum wallboards in the bathroom. Each sample was taken from an approximate surface area of 1 cm^2^ using a sterile cotton tip in a screw-cap plastic tube (Q-tips) or by scraping off small pieces of infected surface materials. The collected samples were kept in tubes in a ventilated room at 10 °C until analysis.

Settled dust samples were collected from all available surfaces (shelves, tables, fridge, tops of the hanging lamps) and other places (excluding the floor) that were regularly cleaned. Each sample was taken from an approximate surface area of 45 × 45 cm using a clean precision Kimwipes^®^ Lite wipe (Kimberly-Clark, GA, USA). Dust was collected both in the bathroom where the water damage occurred and in neighbouring rooms. The collected dust together with the wipes were placed in 50-mL falcon tubes (VWR, Philadelphia, USA), and the tubes were closed and kept in a ventilated room at 10 °C until analysis.

Tape preparations for phase-contrast microscopy (×200 and ×400) were taken directly from the mould-infected area. This was done by gently pressing transparent adhesive tape to the infected surface and mounting it on a microscope slide in a drop of Shear’s mounting fluid [18]. Scrapings were used for fungal identification by metabolite profiling [9] where obtained metabolite profiles from scraped contaminated material were compared to metabolite profiles of in-house indoor *Stachybotrys* strains. Four *S. chartarum* (two chemotype S and two chemotype A) and two *S. chlorohalonata* strains were used for metabolite profiling (Table 1). Agar media used for pure strain inoculation were malt extract agar (MEA) and potato dextrose agar (PDA) [18]. After inoculation, the strains were incubated in darkness at 25 °C for 1–2 weeks, after which sampling for metabolite profiling was performed (PDA and MEA extracts).

**Table 1:** *Stachybotrys* species and strains used in the study for metabolite profiling and comparison

Genus	Species	IBT no.	Chemotype	Origin
*Stachybotrys*	*chartarum*	7709	Macrocyclic trichothecene producer (S)	Building material, DK
*Stachybotrys*	*chartarum*	9631	Macrocyclic trichothecene producer (S)	Home, USA
*Stachybotrys*	*chartarum*	7617	Atranone producer (A)	DK
*Stachybotrys*	*chartarum*	9466	Atranone producer (A)	Gypsum, DK
*Stachybotrys*	*chlorohalonata*	40285	Atranone producer (A)	USA
*Stachybotrys*	*chlorohalonata*	40295	Atranone producer (A)	USA

IBT culture collection, author’s address

### Extraction of pure cultures, biomass and dust

Extraction of pure agar cultures was performed using a micro-scale extraction method modified for *Stachybotrys* metabolites [38]. Three agar plugs (6 mm ID) were cut from a 15-day-old colony from each agar medium (PDA or MEA) and placed in a 2-mL screw-top vial. 1.0 mL of extraction solvent (ethyl acetate/dichloromethane/methanol (3:2:1, *v*/*v*/*v*) containing 1 % formic acid) was added to each vial, and the plugs were extracted by sonication for 60 min. The extracts were further treated as described below for other types of samples.

For fungal biomass collected with Q-tips from the bathroom, the cotton tip of a swab was carefully cut using a disposable scalpel and transferred to 15-mL falcon tube. For the settled dust collected on wipes, the wipes were transferred to 50-mL falcon tubes.

To each Falcon tube was added either 15 mL (dust samples) or 2 mL (biomass samples) extraction solvent (acetonitrile:water (75:25 *v*/*v*) containing 1 % formic acid). The tubes were placed in an ultrasonication bath for 60 min and then centrifuged at 4000*g* for 2 min after which the liquid was transferred to a clean tube and evaporated to dryness under a gentle stream of N_2_. Thereafter, the samples were re-dissolved in 400 μL solvent (acetonitrile:Milli-Q water (75:25 *v*/*v*) with 1 % formic acid) and centrifuged (15 min, 15,000*g*). The supernatant was used directly for chemical analysis, which was conducted within 2 days, where the samples were stored at −20 °C prior to transfer to the autosampler held at 5 °C.

### UHPLC-DAD-QTOF analysis

Metabolite profiling of extracts of pure cultures, biomass and dust samples collected in the water-damaged kindergarten was performed using ultra-high performance liquid chromatography-diode array detection-quadrupole time-of-flight mass spectrometry (UHPLC-DAD-QTOFMS) as described by Dosen et al. [39] using an Agilent Infinity 1290 UHPLC system (Agilent Technologies, Santa Clara, CA, USA) coupled to an Agilent 6545 QTOF MS equipped with a Dual Jet Stream electrospray ion source. The system was equipped with a diode array detector scanning in the range 200–640 nm 20 times/s [36]. Separation was performed on an Agilent Poroshell 120 Phenyl-Hexyl column (2.1 × 150 mm, 2.7 μm) at 60 °C at a flow of 0.35 mL/min. A linear solvent gradient, consisting of A: 20 mmol/L formic acid in water and B: 20 mmol/L formic acid in acetonitrile was used, graduating from 10 to 100 % B within 15 min, held for 2 min, followed by returning to 10 % in 0.1 min and remaining for 3 min, giving a total analysis time of 20 min [36]. Lock mass solution in 80/20 methanol/water (*v*/*v*) was infused in the second sprayer using an extra LC pump at a flow of 1.5 mL/min which was subsequently split 1:100, delivering 15 μL to the MS of 10 μM hexakis(2,2,3,3-tetrafluoropropoxy)phosphazene (Apollo Scientific Ltd, Cheshire, UK) as lock mass. Other MS parameters, including information on automated data-dependant MS/HRMS (auto-MS/HRMS), can be found in [36]. Samples were largely analysed in ESI^+^ mode.

Identification of secondary metabolites was performed using a combination of the following approaches: (i) direct search and matching of MS/HRMS data in the MS/HRMS library and (ii) aggressive dereplication of the full HRMS data where searching was performed using lists of possible known compounds that have been described in the literature but not available as standards. The MS/HRMS library containing ~1500 compounds [36] included spectra from 25 reference standards originating from *Stachybotrys*, a further five tentatively identified *Stachybotrys* compounds and finally one tentatively identified in this study (stachybotrydial). A 300-compound version of this library containing all *Stachybotrys* compounds is available at our WWW site [40].

Aggressive dereplication of the full HRMS data was created by extracting the Antibase2012 database for all compounds having *Stachybotrys* spp. as a source as well as recent references (~140 compounds) [10, 12, 22–24]. Adducts and common fragments included in this search function were [M+H]^+^, [M+Na]^+^, [M+H−H_2_O]^+^ and [M+NH_4_]^+^. All analysed ions were treated as being single charged; the area cut-off was set to 10,000, and the mass spectrum was recorded below 10 % of the peak maximum in order to avoid overloading the detector [36].

For fast screening of a larger number of samples, MassHunter Quantitative Analysis for QTOF (version B.06.00) was used [41]. The method for screening included all *Stachybotrys* compounds with known retention time, selecting the most abundant ion.

### Transferring the QTOF method to QqQ

Analysis of selected *Stachybotrys* metabolites was performed on the Agilent Infinity 1290 UHPLC system coupled to an Agilent 6490 Triple Quadrupole (QqQ) mass spectrometer. Chromatographic separation was performed on an Agilent Poroshell 120 Phenyl-Hexyl column (2.1 × 100 mm, 2.7 μm) held at a temperature of 40 °C and at a flow of 0.4 mL/min. Both eluents contained 20 mM formic acid in water (eluent A) and in acetonitrile/2-propanol (80/20, *v*/*v*) (eluent B). Additionally, 5 mM ammonium formate was added to eluent A. The total analysis time was 10 min, graduating from 20 to 80 % B within 7 min, increasing to 100 % B in 0.1 min, held for 1.1 min, returning to 20 % B in 0.1 min and maintaining until the end of the run. To avoid carry-over, the autosampler was operated in flow-through-needle mode and further coupled to an Agilent Flex cube which was used to perform series of flushing switching between main pass and bypass prior and after the injection at the flow of 4 mL/min. Precursor, product ion, fragmentor voltage and collision energy selection were based on the existing data previously obtained on the QTOF and entered in the MassHunter Personal Compound Database and Library (PCDL) Manager. This library contained information on fragmentation using 10, 20 and 40 eV for each compound. After the initial selection of the precursor and product ion for each transition, the values for collision energies were further optimized around the selected value in order to achieve maximum sensitivity.

The fragmentor voltage was set to 380 V for all compounds/transitions. MassHunter Data Acquisition software version B06.01 was used to control the instrument. All analyses were performed only in ESI^+^ mode using multiple reaction monitoring (MRM) acquisition. For each compound, two or three mass transitions were monitored with the dwell time set to 50 and cycle time of 1605 ms. General source settings were gas temperature of 180 °C, gas flow of 12 L/min, sheath gas temperature of 350 °C, sheath gas flow of 12 L/min, nebulizer 20 psi (137.9 kPa), capillary voltage of 3500 V and nozzle voltage of 0 V in positive mode.

The screening method for metabolites produced by indoor species other than *Stachybotrys* spp. was based on the work of Varga et al. [42]. Two mass transitions per compound, for nine compounds in total, were adopted and further adjusted for our instrument (Electronic Supplementary Material (ESM) Table S1).

### QqQ method validation

For evaluation of method performance, external calibration of four quantitatively available standards (satratoxin H, roridin E, stachybotrylactam and trichodermin) was performed as described in “Chemicals and standards”. Linear non-weighed (satratoxin H, roridin E, stachybotrylactam) or 1/*x* weighed (trichodermin) calibration curves were calculated by plotting the peak area of the analyte signal against the analyte concentration using Agilent MassHunter Quantitative Analysis for QqQ (version B.06.00). Limits of detection (LODs) and lower limits of quantification (LLOQs) were calculated at the lowest concentration levels of liquid standards as concentrations corresponding to a signal-to-noise ratio (*S*/*N*) of 3/1 for LOD and 10/1 for LLOQ. *S*/*N* was calculated using the “Calculate S/N ratio” function in MassHunter Qualitative Analysis. The matrix effect was evaluated for dust samples, by extracting clean, unused Kimwipes^®^ Lite wipes as described in “Extraction of pure cultures, biomass and dust”. Blank extracts (solvents only) and wipes were spiked with a mixture of four standards in triplicates on five (satratoxin H, roridin E) or six (stachybotrylactam, trichodermin) concentration levels and analysed together with the blank extract as a control. This approach enabled direct determination of the matrix effect as the result of signal enhancement/suppression (SSE), by calculating slope ratios of the linear calibration functions SSE(%) = [slope_matrix ‐ matched standards_/slope_liquid standards_] × 100.

## Results and discussion

### Chemical analysis by UHPLC-QTOF

Chemical analysis of extracts of fungal biomass (ESM, Table S2) collected from the kindergarten’s gypsum wallboard showed the presence of both atranones and macrocyclic trichothecenes. Further comparison of these extracts to metabolite profiles from *Stachybotrys* pure agar cultures confirmed the presence of both chemotypes. Whilst these results clearly suggested *S. chartarum* chemotype S as the macrocyclic trichothecene-producing contaminant, identification of the atranone-producing contaminant was more difficult, as chemical analysis could not distinguish between *S. chartarum* chemotype A and *S. chlorohalonata.* Species-level identification of the atranone-producing contaminant would require isolation of the strain and classical morphological methods [9, 18], which were not performed in this study due to the time limitations.

**Fig. 2:**
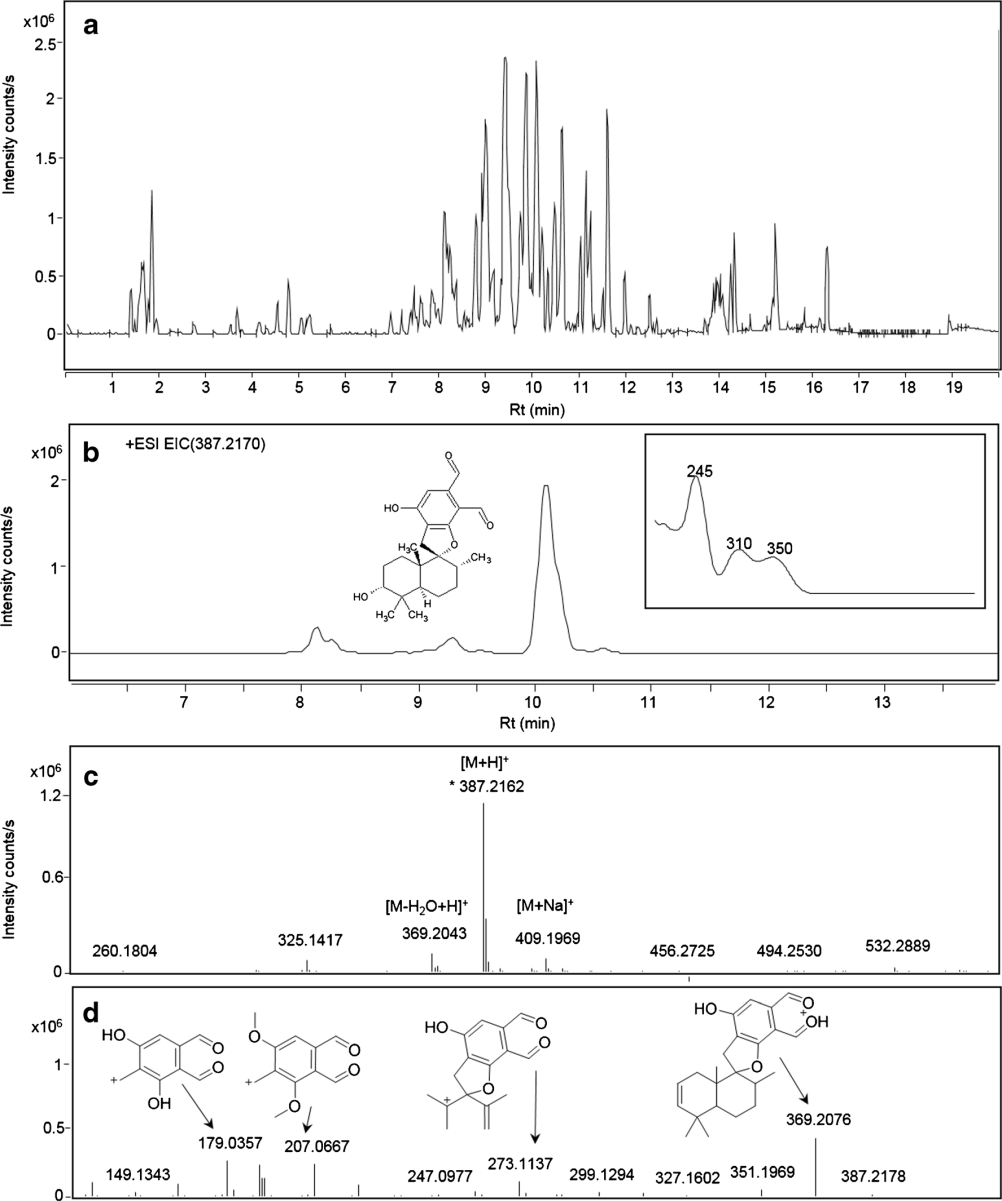
Example of dereplication of stachybotrydial from data obtained on the QTOF: **a** base peak chromatogram (BPC) of *S. chartarum* pure culture (IBT 9631 on MEA) extract, **b** extracted ion chromatogram (EIC) for the *m*/*z* of 387.2166 with UV spectrum characteristic of stachybotrydial [31], **c** full scan spectrum, **d** MS/HRMS of stachybotrydial at 20 eV

Analysis of the gypsum wallboard extracts primarily revealed the presence of spirocyclic drimanes in all the analysed samples. Stachybotrydial and its reduced analogue Mer-NF-5003-B (Fig. 1) were predominant in all analysed extracts. An example of stachybotrydial dereplication is presented in Fig. 2. The observed accurate mass (*m*/*z* 387.2170, 1 ppm deviation) of the peak eluting at 10.1 min perfectly corresponded to the theoretical *m*/*z* for the stachybotrydial molecular ion (387.2166), whose identity was further confirmed with the known UV spectrum for this compound [31, 42]. Furthermore, a full scan spectrum showed the presence of both the sodium adduct [M+Na]^+^ and dehydrated fragment [M−H_2_O+H]^+^, which validated the identification of the molecular ion. Finally, the presence of all the proposed fragments (ESM, Fig S1) in the MS/HRMS 20 eV spectrum served as a final confirmation of stachybotrydial’s identity. After confirming its identity, stachybotrydial was added to the library.

**Fig. 3:**
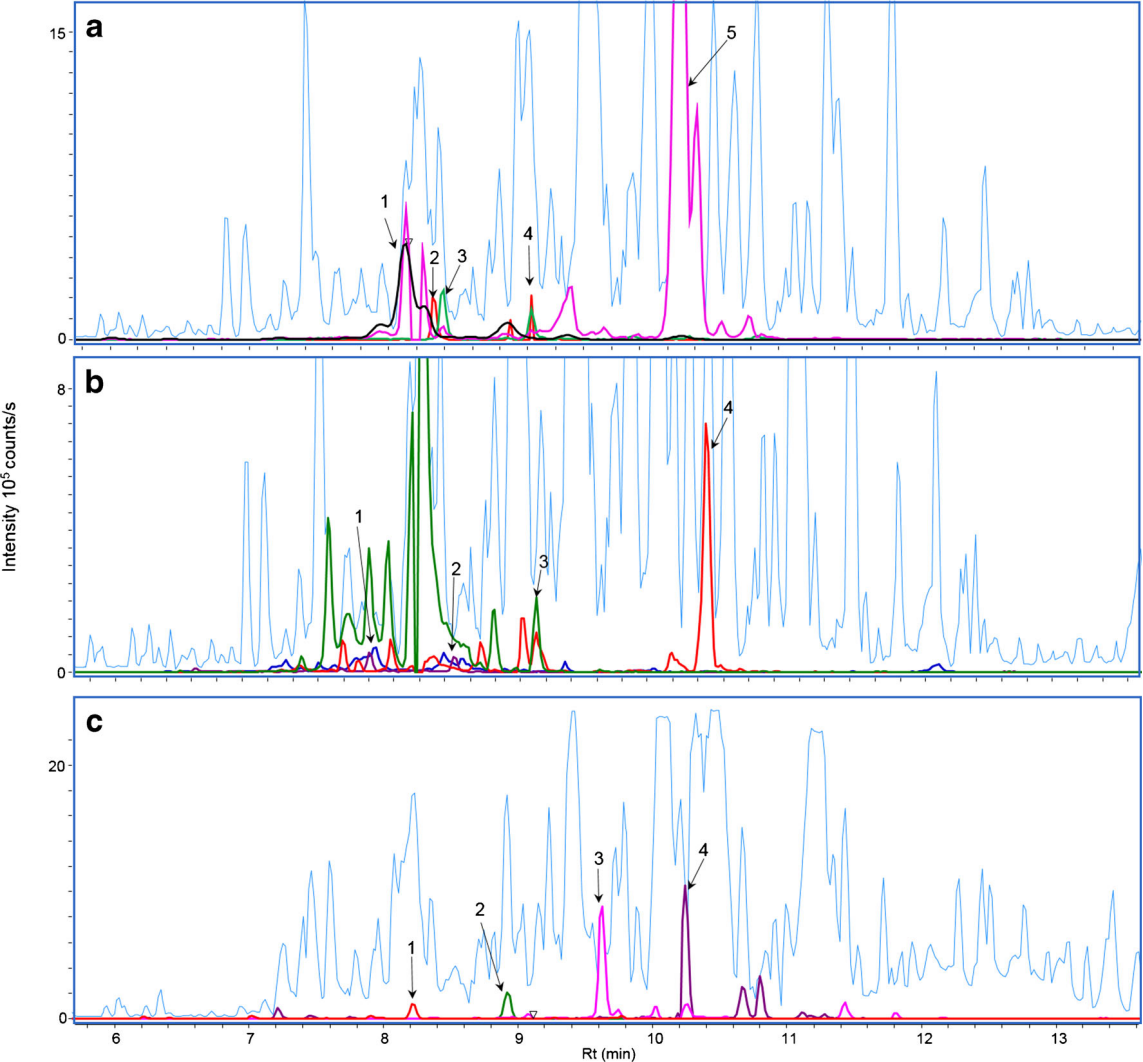
Base peak chromatograms (BPC) of wall scraping (combination of wall swabs 3 and 4) from a contaminated wallboard analysed on QTOF merged with combined extracted ion chromatograms for **a** spirocyclic drimanes: **1** *m*/*z* 405.227 ([M+H]^+^, Mer-NF-5003-B) [43], **2** *m*/*z* 430.2588 ([M+H]^+^, stachybotryamide) [44], **3** *m*/*z* 386.2326 ([M+H]^+^, stachybotrylactam) [42], **4** *m*/*z* 386.2326 ([M+H]^+^, stachybotrylactam isomer) and **5** *m*/*z* 387.2166 ([M+H]^+^, stachybotrydial) [42]; **b** macrocyclic trichothecenes: **1** *m*/*z* 546. 2695 ([M+NH_4_]^+^, satratoxin H), **2** *m*/*z* 562.2645 ([M+NH_4_]^+^ satratoxin G), **3** *m*/*z* 548.2855 ([M+NH_4_]^+^ roridin L2) and **4** *m*/*z* 532.2904 ([M+NH_4_]^+^ roridin E); and **c** diterpenoids and their precursors: **1** *m*/*z* 319.227 ([M+H]^+^,3,4-epoxy-6-hydroxydolabella-7*E*,12-dien-14-one), **2** *m*/*z* 303.2319 ([M+H]^+^, 6-hydroxydolabella-3*E*,7*E*,12-trien-14-one), **3** *m*/*z* 417.227 ([M+H]^+^, atranone A) and **4** *m*/*z* 447.238 ([M+H]^+^, atranone B)

Two peaks with *m*/*z* of 386.2326 (4 ppm deviation) and a retention time (RT) difference of 0.7 min were identified as stachybotrylactam in all extracts. No difference in fragmentation pattern at 10 and 20 eV was found between the two compounds. Comparison with the stachybotrylactam standard identified the peak eluting at 8.35 min as the known compound, whilst a later-eluting compound will henceforth be referred to as the stachybotrylactam isomer. Library matching also identified a peak eluting at 8.1 min (*m*/*z* 405.227, 2.2 ppm deviation) as Mer-NF-5003-B, as well as a peak eluting at 8.3 min (*m*/*z* 430.2588, 1.6 ppm) as stachybotryamide (Fig. 3). Both matches were further confirmed using UV spectra from authentic standards (ESM, Fig S2).

In four out of seven wall scrapings (ESM, Table S2) from the water-damaged bathroom, aggressive dereplication suggested the presence of atranone A and B, as well as (1S*,6S*,11S*)-6-hydroxydolabella-3*E*,7*E*,12-trien-14-one (Fig. 3), which was further confirmed by authentic standards and UV/vis data [14] (ESM, Fig. S3). Furthermore, analysis of wallboard scrapings from the kindergarten not only showed the presence of spirocyclic drimanes and atranones but also yielded a positive hit for satratoxin H, showing intense [M+NH_4_]^+^ and [M+Na]^+^ as well as the MS/HRMS spectrum perfectly matching an authentic standard. Searching MS/HRMS data for *m*/*z* 231.1300 ± 0.0100, a characteristic fragment of trichothecenes [14], confirmed not only the presence of satratoxin H but also the presence of other trichothecenes (roridin L2, satratoxin G, roridin E, Fig S4, ESM), which were also verified by comparison to authentic standards. The compound with an RT of 10.45 min possessed the characteristic fragment with *m*/*z* 231.1384 and also showed the presence of *m*/*z* 361.2003 characteristic of roridin E [14], as well as the precursor ion of *m*/*z* 532.2904 (1.3 ppm deviation) matching [M+NH_4_]^+^ for roridin E. In this sample, the intensities of the trichothecenes with respect to the intensities of the spirocyclic drimanes were significantly (three- to fivefold) lower, increasing the risk of overlooking these compounds in the presence of drimanes. The problem of detection of atranones, dolabellanes and macrocyclic trichothecenes in the presence of spirocyclic drimanes is well known [12, 31]. Jarvis et al. [12] developed a normal phase SPE method (PEI silica) for fractionation of the *S. chartarum* extracts in spirocyclic drimanes and macrocyclic trichothecenes or atranones. Due to the possible loss of the target analyte and time associated with fractionation, we decided to attempt to increase the sensitivity of our method for the low-abundant compounds by transferring the method to the more sensitive and selective UHPLC-QqQ.

### UHPLC-QTOF to UHPLC-QqQ method transfer

In contrast to the UHPLC-QTOF method, the UHPLC-QqQ gradient was started at 20 % organic phase (eluent B) instead of 10 %, a shorter column was used and there was a steeper increase to 100 % acetonitrile/2-propanol (eluent B) to elute compounds faster and as sharper peaks. In order to provide sufficient precursor ion intensity and suppress the formation of [M+Na]^+^ (which gave poor daughter ion yields) for macrocyclic trichothecenes and promote [M+NH_4_]^+^ formation, ammonium formate was added to eluent A. For each macrocyclic trichothecene, both mass transition with molecular ion [M+H]^+^ and ammonium adduct [M+NH_4_]^+^ as precursor ion were used, thus ensuring reliable identification (Table 2). Mass transition giving the most abundant signal was chosen as the quantifier ion, regardless of whether the chosen precursor was the proton adduct [M+H]^+^ or ammonium adduct [M+NH_4_]^+^. Since all the compounds included in the method eluted between 3 and 5.5 min, the retention time window for all transitions was set to 4 min (from 2 to 6 min). With the dwell time for each transition of 50 ms, the duty cycle of 1605 ms and the typical peak width of 20 s, we obtained a minimum of 12 data points across peak, which was enough to ensure reliable peak integration [45].

**Table 2:** List of *Stachybotrys* metabolites included in QqQ method with optimized ESI-MS/MS

Metabolite	Rt (min)	*m*/*z* precursor ion	Ion species	*m*/*z* product ion (collision energy (V))
Satratoxin H^a^	3.60	546	[M+NH_4_]^+^	249(15)/231(15)
		**529**	[M+H]^+^	**231(15)**
Satratoxin G^a^	3.50	562	[M+NH_4_]^+^	249(15)/231(15)
		**545**	[M+H]^+^	**231(15)**
Roridin L2^a^	4.20	548	[M+NH_4_]^+^	249(15)/231(15)
		**531**	[M+H]^+^	**231(15)**
Roridin E^a^	5.10	**532**	[M+NH_4_]^+^	**361(15)**/231(15)
		515	[M+H]^+^	231(15)
Atranone A^b^	4.60	417	[M+H]^+^	381(10)/357(15)
Atranone B^b^	5.40	447	[M+H]^+^	387(10)/369(10)
Dolabellane^a, b, c^	3.90	303	[M+H]^+^	219(15)
Stachybotrylactam^a^	4.10	**386**	[M+H]^+^	256(20)/**178(35)**/150(40)
Stachybotrylactam isomer	3.70	**386**	[M+H]^+^	256(20)/**178(35)**
Stachybotryamide	4.10	**430**	[M+H]^+^	**260(30)**/222(20)
Stachybotrydial	4.90	**387**	[M+H]^+^	207(20)/**179(20)**
Mer-NF-5003-B	3.65	**405**	[M+H]^+^	387(15)/**369(20)**
Trichodermin^a^	3.85	**293**	[M+H]^+^	143(10)/**109(15)**

Precursor ion-to-quantifier ion mass transition together with collision energy used is presented in bold

^a^Reference standard available

^b^Not quantified due to the unavailability of the quantitative standard with the same/similar structure

^c^(1S*,3R*,4R*,6S*,11S*)-3,4-Epoxy-6-hydroxydolabella-7*E*,12-dien-14-one

Collision energies, chosen through comparison with the QTOF MS/HRMS spectra obtained at 10, 20 and 40 eV, only required minimal optimization (changes of less than 5 eV) in most cases. This demonstrates the value of the information that can be obtained from full scan QTOF experiments, as well as that acceptable QqQ methods can be obtained by direct library-to-QqQ transfer.

Transitions and starting collision energies for chosen metabolites included in the method detecting secondary metabolites produced by indoor fungi other than *Stachybotrys* were adopted from Varga et al. [42]. Further optimization of this method only included collision energy adjustment within a few volts (ESM, Table S1). The fragmentor voltage (380 V) cannot be altered between compounds as it is fixed by the ion-funnel instrument, but far less important than on a cone-based instrument.

### UHPLC-QqQ method validation

The matrix effect was calculated as the slope ratio of the linear functions for matrix-matched standards and liquid standards and is presented in Table 3. The matrix effect was evaluated only for one matrix, clean Kimwipes^®^ Lite wipes used to collect dust. This matrix was chosen due to the fact that dust needed to be extracted together with the wipes. Among evaluated metabolites, both satratoxin H and roridin E showed enhancement of the signal, which was minor for roridin E (4 %) and significantly higher for satratoxin H (34 %). In contrast, signals for stachybotrylactam and trichodermin were suppressed by 11 and 21 % respectively. Evaluation of blank matrix extract showed no signal for any of the tested analytes. Clearly, it can be expected that dust itself will further suppress/enhance the signals obtained; however, one should note that the dust samples taken were very low and taken from places cleaned several times per week, and not behind furniture or other places rarely cleaned. Vishwanth et al. [26] reported a severe matrix effect in dust for the majority of the target analytes; however, the reported enhancement for stachybotrylactam by 6 % was negligible, whilst the matrix effect for roridin E, satratoxin H and trichodermin was not reported. Undoubtedly, the observed enhancement and suppression in our study showed that matrix-matched calibration is not adequate to account for the matrix effect and that the use of isotopic-labelled internal standards would be preferable [26, 42].

**Table 3:** Matrix effect, signal suppression/enhancement (SSE), standard error of regression line (Sy), average observed accuracy (Acc) and maximum observed accuracy (Acc_max_), coefficients of variation (CV), limit of detection (LOD) and lower limit of quantification (LLOQ) values for *Stachybotrys* metabolites

Metabolite	SSE^d^ (%)	Sy^e^	Acc (Acc_max_)^f^ (%)	CV^g^ (%)	LOD^h^ (ng/cm^2^)	LLOQ^i^ (ng/cm^2^)
Satratoxin H^a^	134	1.8	28 (79)	2.2	15	50
Satratoxin G^a^	134	1.8	28 (79)	2.2	15	50
Roridin E^b^	104	4.9	29 (114)	2.2	0.1	0.2
Roridin L2^b^	104	4.9	29 (114)	2.2	0.1	0.2
Stachybotrylactam^c^	89	36	21 (77)	2.5	2	6
Stachybotrylactam isomer^c^	89	36	21 (77)	2.5	2	6
Stachybotryamide^c^	89	36	21 (77)	2.5	2	6
Stachybotrydial^c^	89	36	21 (77)	2.5	2	6
Mer-NF-5003-B^c^	89	36	21 (77)	2.5	2	6
Trichodermin	79	40	13.4 (28)	4.4	5	17

^a^Compounds calibrated against satratoxin H standard

^b^Compounds calibrated against roridin E standard

^c^Compounds calibrated against stachybotrylactam standard

^d^SSE%—signal suppression/enhancement calculated as the slope ratio of the linear functions for matrix-matched standards and liquid standards multiplied by 100

^e^Standard error of the regression line

^f^Average accuracy on all levels—accuracy on each level was calculated as the ratio of the calculated concentration and the expected concentration multiplied by 100. Acc_max_ represents the maximum observed value regardless of the level including the values outside allowed limits (20 %)

^g^CV%—calculated as the average of the ratios of the standard deviation and average concentration multiplied by 100 for each level

^h^LOD—the limit of detection calculated at the lowest concentration levels as concentrations corresponding to a signal-to-noise ratio (*S*/*N*) 3/1

^i^LLOQ—lower limit of quantification calculated at the lowest concentration levels as concentrations corresponding to a signal-to-noise ratio (*S*/*N*) 10/1

The linear range covered three orders of magnitude for stachybotrylactam and trichodermin, but only two orders of magnitude for satratoxin H and roridin E, due to the limited quantities of these two standards. All constructed calibration curves showed good linearity across the entire range, and given the expected amounts in dust, this is considered sufficient. Calculated coefficients of variation (CV %) for both liquid and matrix-matched standards were generally low, with the highest for trichodermin (8 % for liquid standards and 4 % for matrix-matched standards). Non-weighed calibration curves were chosen for stachybotrylactam, roridin E and satratoxin H, due to the higher resulting linearity and the fact that the concentrations in analysed samples tended to shift towards the middle and higher ranges of the constructed curves. Use of non-weighed calibration curves resulted in accuracy outside of the allowed limits (20 %) on the lowest level; however, the observed accuracy on all other levels was within limits (Table 3). It should be noted that dust was sampled and analysed on a much larger surface (45 × 45 cm) and later recalculated to the amount per square centimetre; therefore, the obtained concentrations in all samples are comparable. In the absence of quantitative standards for all mycotoxins included in the method, stachybotryamide, stachybotrydial and Mer-NF-5003-B were quantified based on the calibration curve for stachybotrylactam. Similarly, roridin L2 was quantified based on the calibration curve constructed for roridin E, whilst satratoxin G was quantified based on the calibration curve for satratoxin H. This approach was obviously based on similarity in structures, although we are well aware that similar structures often show differences in fragmentation patterns and intensities of created fragments. Although possessing different elemental compositions and thereby different precursor ions, all macrocyclic trichothecenes produce same fragments, namely *m*/*z* 231 and 249. Only roridin E exhibits specific mass transition (*m*/*z* 532 to 361), which was chosen for quantification.

The situation was more complicated in the case of the spirocyclic drimanes, where the structural differences between compounds were greater, most notably in the lack of 2-pyrrolidone moiety in stachybotrydial and Mer-NF-5003-B in comparison to stachybotrylactam and stachybotryamide (Fig. 1). Clearly, calculated concentrations, especially in cases where structural differences between metabolites were more prominent, are likely to be more inaccurate; however, since reference standards for all drimanes were not commercially available, we found that it was a better option to calibrate against stachybotrylactam which is commercially available.

### Wall swabs and dust wipe analyses by QqQ

The UHPLC-QqQ method was used to analyse dust wipes and wall swabs collected in the water-damaged kindergarten. The method was able to detect *Stachybotrys* compounds in settled dust, both in the infected bathroom and in the adjacent rooms, and further differentiate two co-existing chemotypes. The results showing all detected metabolites are presented in Table 4.

**Table 4:** Estimated amounts (single sample, single injection) of *Stachybotrys* metabolites found on contaminated wall surface and in settled dust

Samples	Concentration (pg/cm^2^)								Area		
	Stachybotryamide	Stachybotrylactam	Stachybotrylactam isomer	Stachybotrydial	Satratoxin H	Satratoxin G	Roridin L2	Roridin E	Atranone A^d^	Atranone B^d^	Dolabellanes^d^
Wall swab 1	18 × 10^4^	32 × 10^4^	35 × 10^4^	37 × 10^4^	ND	ND	ND	ND	31,166	2578	590,773
Wall swab 2	18 × 10^4^	56 × 10^4^	65 × 10^4^	54 × 10^4^	ND	ND	ND	ND	57,702	3104	775,107
Wall swab 3	18 × 10^4^	36 × 10^4^	55 × 10^4^	23 × 10^4^	18 × 10^4^	ND	37 × 10^3^	30 × 10^3^	8695	1481	187,816
Wall swab 4	18 × 10^4^	104 × 10^4^	91 × 10^4^	38 × 10^4^	ND	14 × 10^4^	55 × 10^3^	13 × 10^4^	ND	ND	ND
Wall swab 5	18 × 10^4^	38 × 10^4^	40 × 10^4^	32 × 10^4^	ND	ND	ND	ND	33,072	1096	607,685
Wall swab 6	ND	14 × 10^4^	14 × 10^4^	ND	ND	ND	ND	ND	ND	ND	ND
Wall swab 7	17 × 10^4^	16 × 10^4^	18 × 10^4^	17 × 10^4^	14 × 10^4^	ND	35 × 10^3^	17 × 10^3^	4926	380	5381
Dust wipe 1^a, b^	90	230	180	100	ND	ND	ND	10	18,467	ND	495,672
Dust wipe 2^a, c^	80	90	90	80	ND	ND	ND	ND	2718	ND	25,308
Dust wipe 3^a, c^	ND	70	70	ND	ND	ND	ND	ND	ND	ND	ND
Dust wipe 4^a, c^	ND	70	70	ND	ND	ND	ND	ND	ND	ND	ND
Dust wipe 5^a, c^	ND	ND	70	ND	ND	ND	ND	ND	ND	ND	ND
Dust wipe 6^a, c^	ND	70	70	ND	ND	ND	ND	6.4	ND	ND	ND
Dust wipe 7^a, c^	ND	70	70	ND	ND	ND	ND	ND	ND	ND	ND
Dust wipe 8^a, c^	ND	70	70	ND	ND	ND	ND	ND	ND	ND	ND
Dust wipe 9^a, c^	ND	70	70	ND	ND	ND	ND	ND	ND	ND	ND
Dust wipe 10^a, c^	ND	70	70	ND	ND	ND	ND	ND	ND	ND	ND
Dust wipe 11^a, c^	ND	70	70	ND	ND	ND	ND	ND	ND	ND	ND

^a^Concentrations found in dust samples were analysed for the entire sampled surface (2025 cm^2^) and recalculated to amounts per square centimetre

^b^Dust sampled in the room where water damage occurred

^c^Dust sampled in rooms adjacent to the room where water damage occurred

^d^Metabolites only identified and not quantified due to the lack of quantitative standards with the same/similar structure

The results confirmed the presence of both macrocyclic trichothecene- (chemotype S) and atranone-producing contaminants (chemotype A) in both wall and dust samples. In two out of seven wall swab samples, both trichothecene mycotoxins and atranones/dolabellanes were detected in the same sample (wallboard swabs 3 and 7). In other cases, there was either trichothecenes or atranones/dolabellanes present in the wall swabs, depending on the sampling site on the wallboard. This shows the coexistence of two chemotypes on the same material, with no clear borderline between areas of their contamination. Among trichothecenes, the highest amounts (180 ng/cm^2^) were estimated for satratoxin H, whilst atranones/dolabellanes could not be quantified due to the insufficient amounts of the standard. No trichodermin was found either in dust wipe or wall swab samples. Spirocyclic drimanes represented by stachybotrylactam, stachybotrydial and stachybotryamide were dominant in all samples as expected, based on results obtained on the QTOF. The exception was Mer-NF-5003-B, whose production on the materials was reduced compared to pure cultures. Estimated concentrations for drimanes in samples collected with Q-tips were within one order of magnitude (135–1042 ng/cm^2^) with the total amount for all drimanes collected with the Q-tip estimated to be 2.5 μg/cm^2^. Screening for metabolites produced by species other than *Stachybotrys* revealed the presence of roquefortine C and meleagrin. This suggests the presence of *Penicillium chrysogenum* on sampled material, which is among species most commonly isolated from indoor environment [46].

**Fig. 4:**
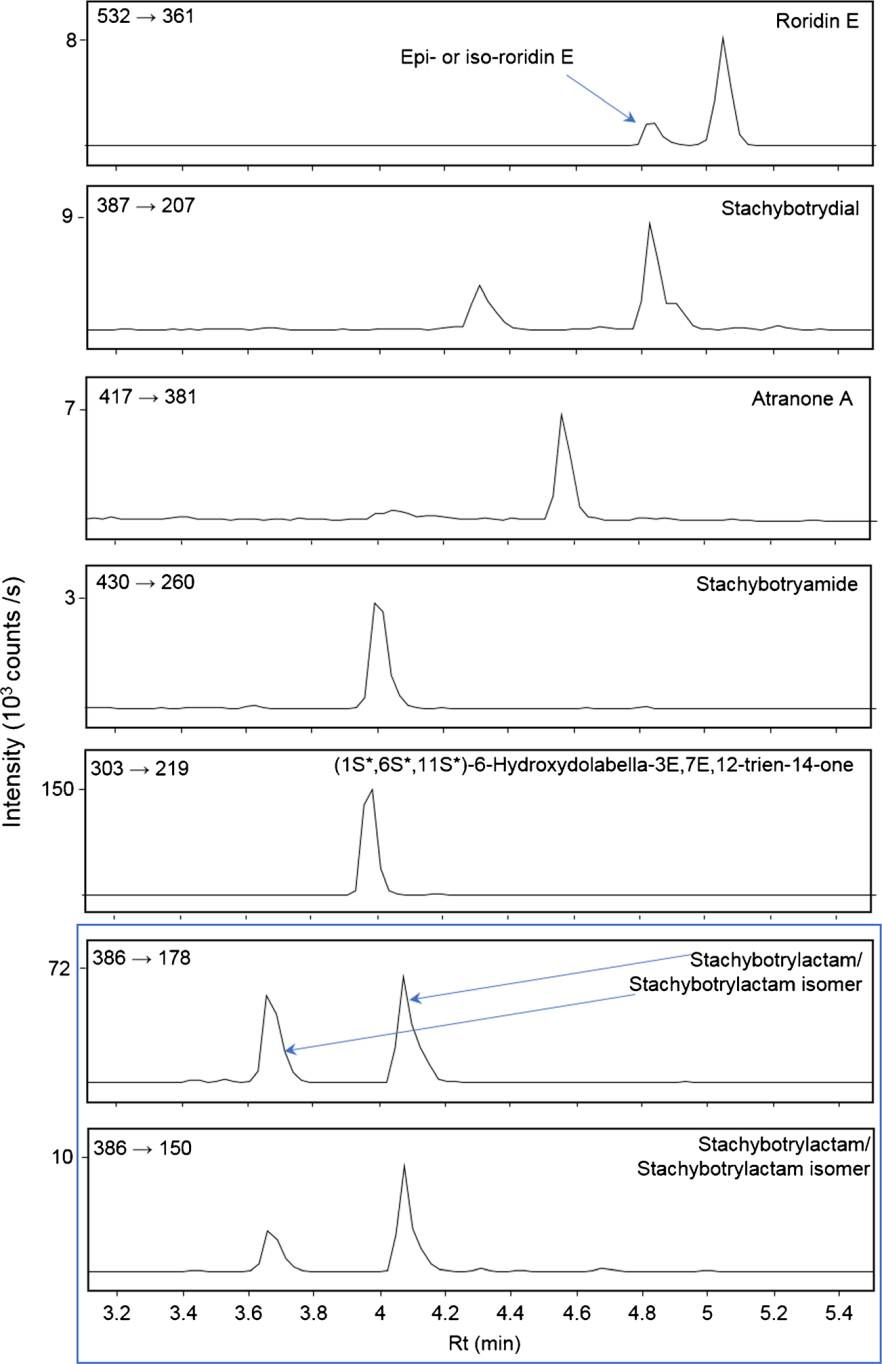
MS/MS transitions for all mycotoxins identified in dust sample collected in the water-damaged room

For the dust wipe samples, all groups of metabolites (trichothecenes, atranones/dolabellanes, drimanes) were detected in settled dust collected in the bathroom where water damage occurred (dust 1, Fig. 4). Settled dust collected in adjacent rooms contained, in most cases, stachybotrylactam and stachybotrylactam isomer. Roridin E was found only in one sample in the concentration of 6.4 pg/cm^2^, whilst dolabellanes were found in two samples; however, the peaks were of very low intensity. Neither meleagrin nor roquefortine C was found in the dust wipe samples. The amounts of mycotoxins in dust wipes in comparison to the amounts found in the wall swabs were three to four orders of magnitude lower, both in the cases of drimanes and trichothecenes (600 pg/cm^2^ of total drimanes in the bathroom with the water damage and highest estimated total amount of drimanes of 340 pg/cm^2^ in the adjacent room). Although stachybotrylactam has been reported in dust samples previously, its presence has only been found in samples collected from waste management facilities [47] and in settled dust collected in Dutch schools (in only 1 % of the collected samples, [48]). In addition to this, attempts have been made to quantify macrocyclic trichothecenes in dust; however, only the simple trichothecenes verrucarol and trichodermol (hydrolysis products of the satratoxins) analysed by GC-MS have been reported [30, 48, 49]. Furthermore, Polizzi et al. [27] reported the analysis of roridin E on contaminated material but not in dust. To the best of our knowledge, this is the first study where macrocyclic trichothecenes have been found directly in settled dust collected from a water-damaged building. It is also the first time that a comparison between the amounts of mycotoxins produced on contaminated wall surfaces and the amounts of mycotoxins that actually become airborne has been made. Moreover, the presence of drimanes in dust samples collected in rooms adjacent to the room with water damage clearly demonstrates a wide distribution of mycotoxins in detectable amounts and this is promising for their use as biomarkers.

The mycotoxin content of settled dust is important for overall exposure assessment. The presented method appeared to be a useful tool for identifying and estimating the amounts of indoor contaminants in dust. This indicates that the future focus could primarily be analysis of dust samples. Given that dust is the primary route of exposure to these mycotoxins, but the detection here has previously proven difficult, this method may provide an effective means of gathering considerable chemical data and improve our understanding of mycotoxin toxicity in contaminated buildings.

## Conclusion

This case study presents a semi-quantitative UHPLC-QqQ method with several new exposure biomarkers, which was developed based on UHPLC-qQTOF screening of culture extracts. The method was able to identify 12 *Stachybotrys* metabolites of which four could be quantified based on authentic standards and an additional six estimated based on similar compounds. The method was applied to samples collected in a water-damaged building contaminated by *S. chartarum* chemotype S, as well as with atranone-producing contaminant (*S. chartarum* chemotype A or *S. chlorohalonata*). The obtained results represent a step forward in solving the problems of exposure to mycotoxins in damp indoor environments, detecting for the first time the presence of the same mycotoxins on contaminated gypsum wallboard surfaces and in settled dust. Furthermore, the method enables fast estimation of the mycotoxin’s amounts in analysed samples. Demonstrating that method transfer from UHPLC-qQTOF to UHPLC-QqQ instruments is facile means that this method can be supplemented with additional biomarkers in subsequent exposure studies. This methodology represents a significant advance in the detection of mycotoxins in dust samples, which is the main route of exposure leading to toxicity and illness. As such, this study could have an impact on our understanding of the relationship between mould exposure and sickness. Further work includes expanding this methodology to include mycotoxins and biomarkers produced by other species and genera of indoor fungi and further case studies to demonstrate the reliability and applicability of this method.

## Electronic supplementary material

Below is the link to the electronic supplementary material.

ESM 1 (PDF 741 KB)
