# Supplementary material for: Stachybotrys mycotoxins: from culture extracts to dust samples
Source: Anal Bioanal Chem. 2016 Jun 2;408:5513–26. doi: 10.1007/s00216-016-9649-y (PMC4939167; doi:10.1007/s00216-016-9649-y)
Supplement: Supplementary file 1 — (PDF 741 KB) [file 216_2016_9649_MOESM1_ESM.pdf]

Analytical and Bioanalytical Chemistry

Electronic Supplementary Material

Stachybotrys mycotoxins: from culture extracts to dust samples

Ina Došen, Birgitte Andersen, Christopher B. W. Phippen, Geo Clausen, Kristian Fog Nielsen

Table S1 List of metabolites produced by species other than *Stachybotrys* spp. included in QqQ method with optimized ESI-MS/MS parameters (adopted from Varga et al., 2013)

Metabolite	Rt (min)	*m/z* precursor ion	Ion species	m/z product ion (collision energy (V))
Altenuene	3.2	293	$[M+H]^+$	275(5)/257(10)
Austdiol	1.6	237	$[M+H]^+$	159(20)/117(25)
Chaetoglobosin A	4.4	529	$[M+H]^+$	511(5)/130(35)
Cytochalasin A		478	$[M+H]^+$	120(25)/91(40)
Meleagrin	3.4	434	$[M+H]^+$	403(10)/334(20)
Mycophenolic acid		338	$[M+NH_4]^+$	303(10)/207(25)
Penicillin G	3.3	335	$[M+H]^+$	176(10)/160(5)
Roquefortine C	4.3	390	$[M+H]^+$	322(15)/193(25)
Sterigmatocystin	4.6	325	$[M+H]^+$	281(40)/130(25)

Table S2 Overview of Stachybotrys metabolites identified on QTOF in pure agar cultures (MEA and PDA) and wallboard scrapings collected in water-damaged kindergarten

	Metabolites											
Samples	Stachybotryamide	Stachybotrylactam	Stachybotrylactam Isomer	Stachybotrydial	Mer-NF-5003-B	Satratoxin H	Satratoxin G	Roridin L2	Roridin E	Atranone A[d]	Atranone B[d]	Dolabellanes[d]
S. chartarum IBT 9631	+	+	+	+	+	+	+	+	+	-	-	-
S. chartarum IBT 7709	+	+	+	+	+	+	+	+	+	-	-	-
S. chartarum IBT 7617	+	+	+	+	+	-	-	-	-	-	-	+
S. chartarum IBT 9466	+	+	+	+	+	-	-	-	-	-	-	+
S. chartarum IBT 40285	+	+	+	+	-	-	-	-	-	+	+	+
S. chartarum IBT 40295	+	+	+	+	-	-	-	-	-	+	+	+
Wall scraping 1	+	+	+	-	+	-	-	-	-	+	+	+
Wall scraping 2	+	+	+	-	+	+	+	+	+	+	+	+
Wall scraping 3	+	+	+	+	+	-	-	-	+	+	+	+
Wall scraping 4	+	+	+	-	-	-	-	-	+	+	-	+
Wall scraping 5	+	+	+	-	-	-	-	-	+	-	-	-
Wall scraping 6	+	+	+	+	+	-	-	-	-	+	+	+
Wall scraping 7	+	+	+	-	+	+	-	+	+	-	-	+

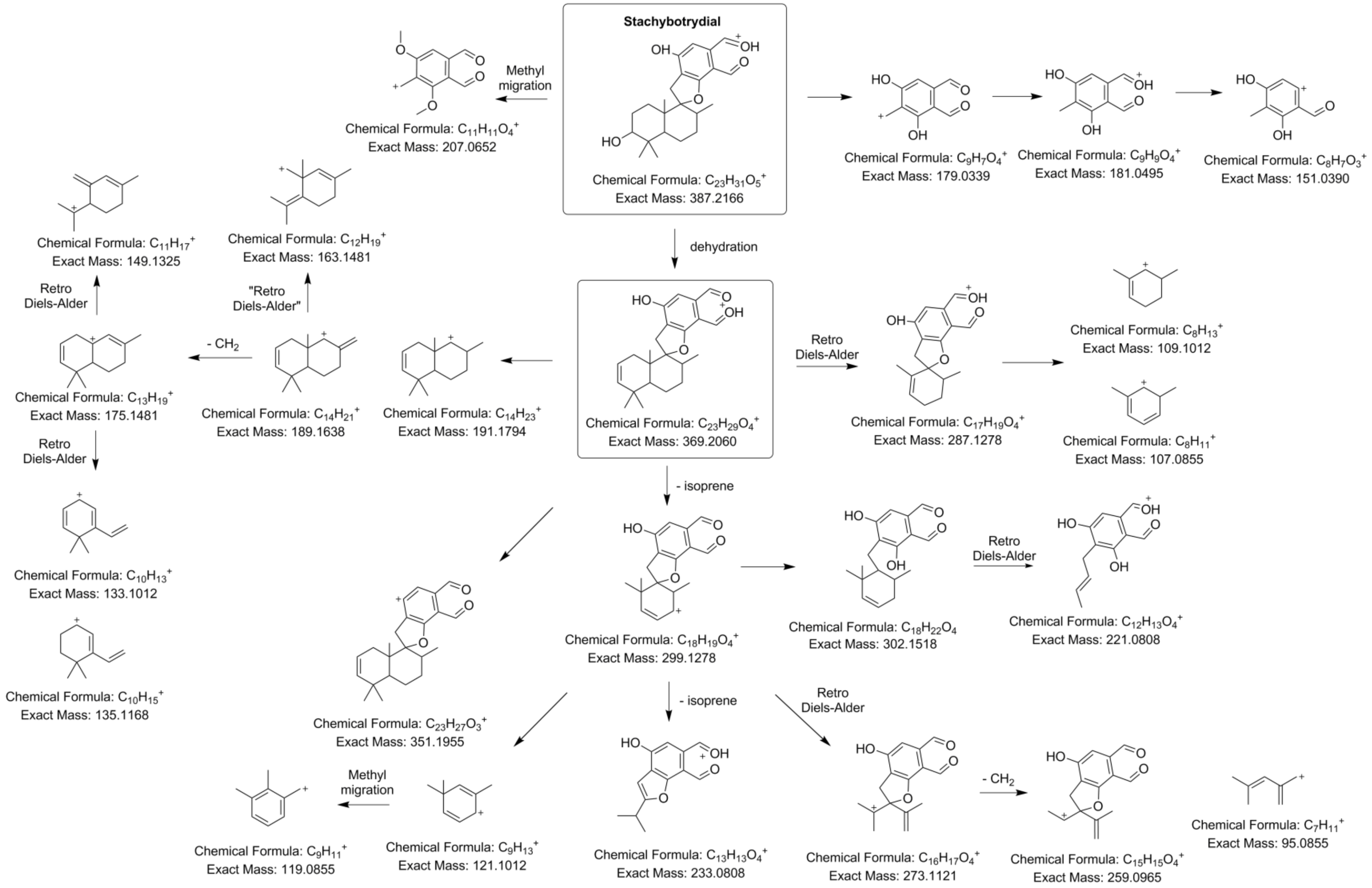

Fig. S1 Proposed fragmentation pattern for stachybotrydial

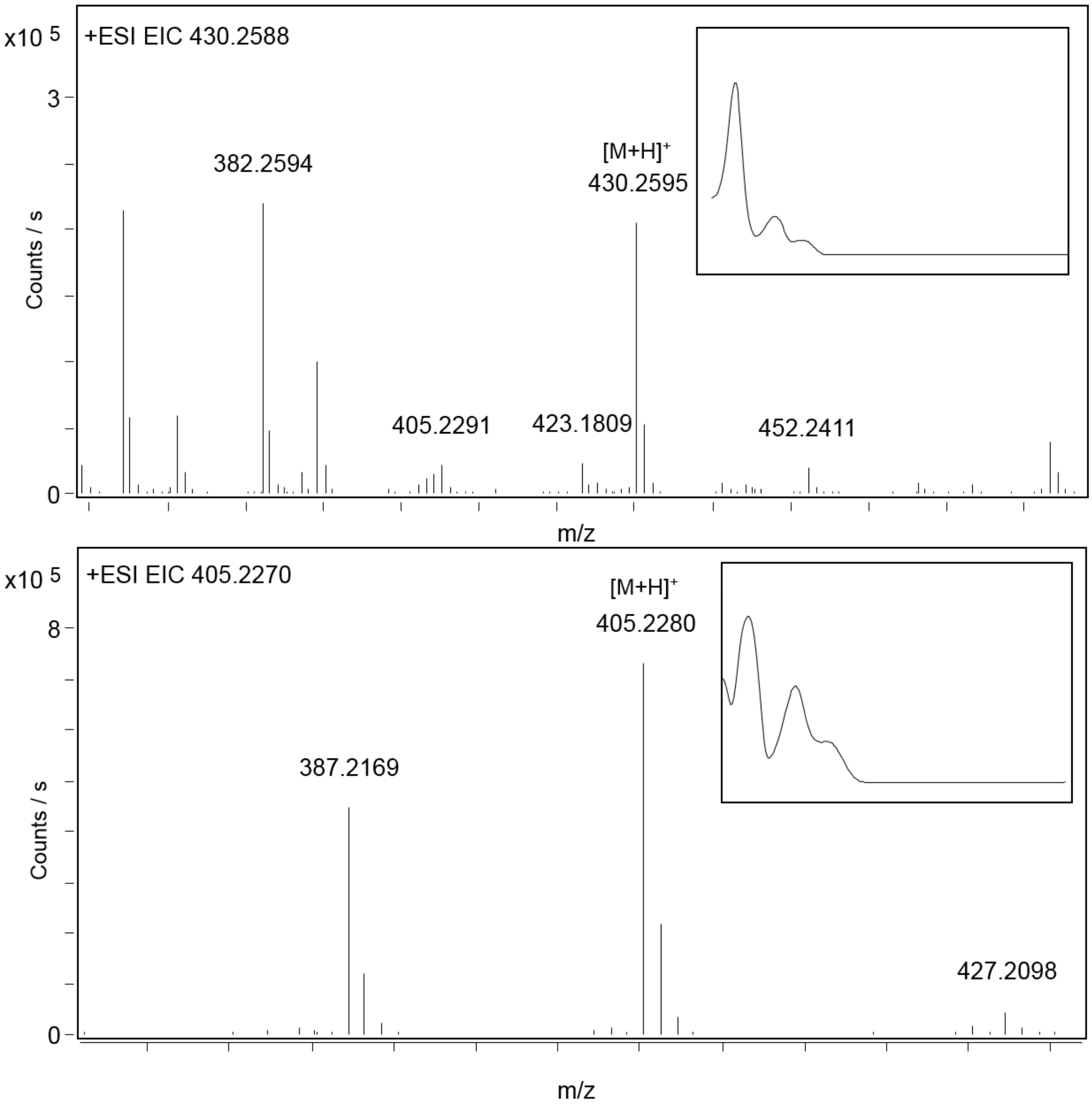

Fig. S2 Corresponding MS spectra of extracted ion chromatographs (EIC) and representative UV spectra for stachybotryamide (*m/z* 430.2588, 4.2 ppm) and Mer-NF-5003-B (*m/z* 405.2270, 2.5 ppm)

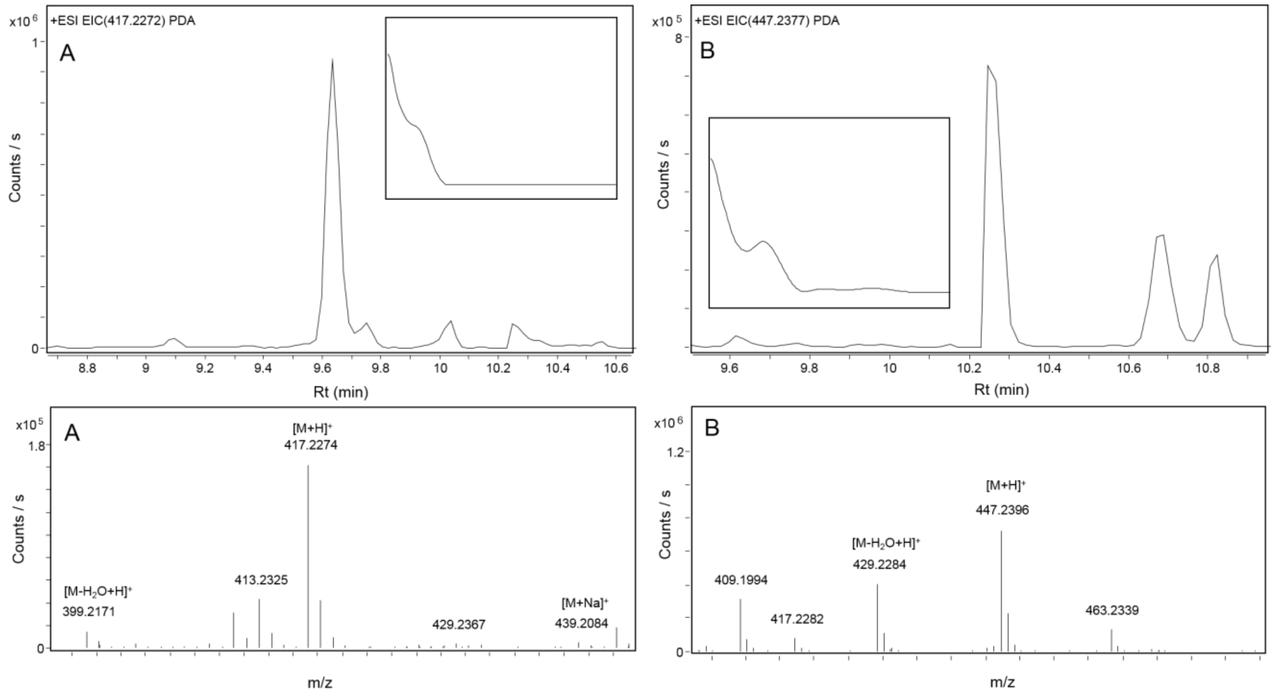

Fig. S3 Extracted ion chromatogram (EIC), full scan spectrum and UV confirmation for: A – atranone A (*m/z* 417.2272, 0.5 ppm), B – atranone B (*m/z* 447.2377, 4.5 ppm)

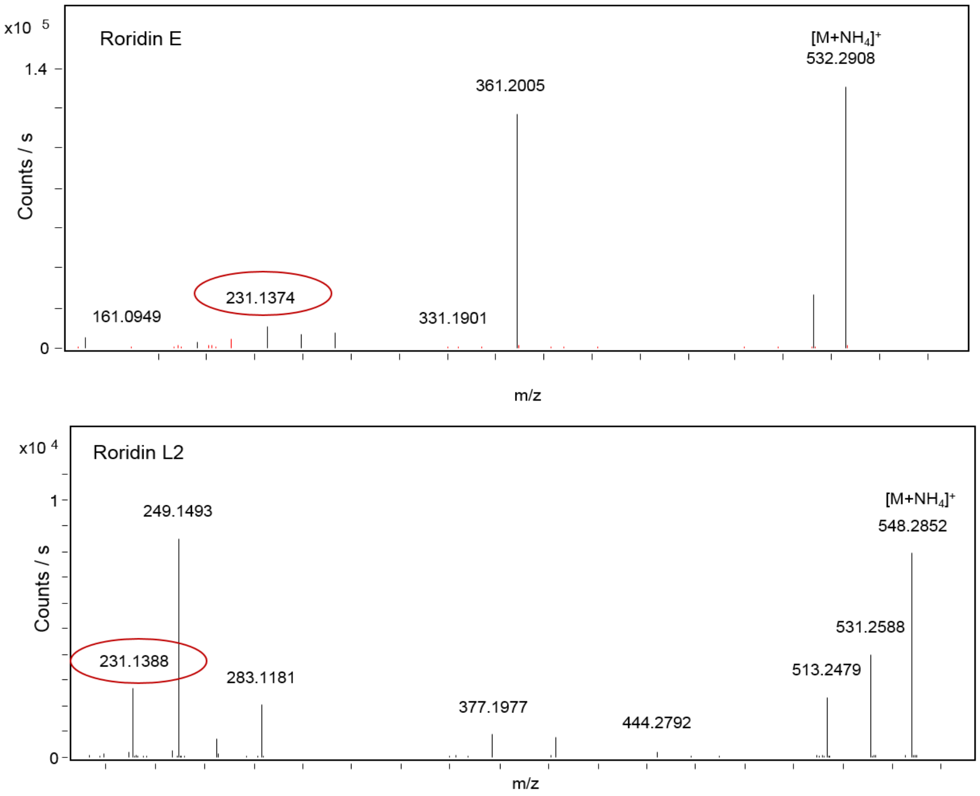

Fig. S4 MS/HRMS of macrocyclic trichothecens (roridin E and roridin L2) found by searching for 231.1300 ± 0.0100 fragment ion
